# Supplementary material for: Asymmetric Synthesis of Quaternary Hydantoins via a Palladium-Catalyzed Aza-Heck Cyclization
Source: J Am Chem Soc. 2025 Nov 14;147(49):44692–8. doi: 10.1021/jacs.5c16022 (PMC12703750; doi:10.1021/jacs.5c16022)
Supplement: Supplementary file 2 [file ja5c16022_si_002.zip › All NMR FID Files/S43/S43_AllNMR/2/pdata/1/email_EM04-044-1_2_1.pdf]

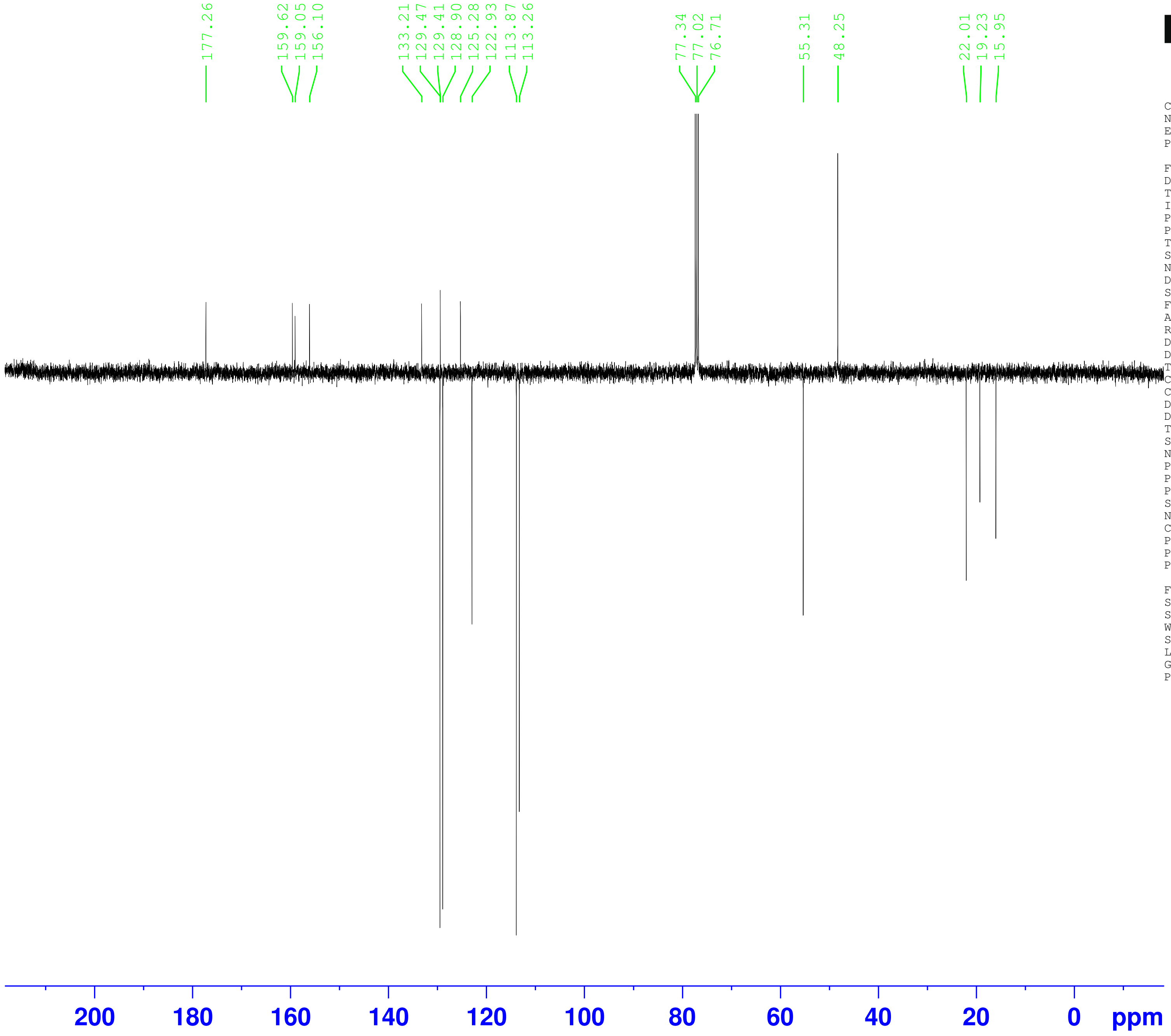

BRUKER

```
Current Data Parameters
NAME            EM04-044-1
EXPNO                    2
PROCNO                   1

F2 - Acquisition Parameters
Date_             20230401
Time                 15.16 h
INSTRUM   Avance Neo 400 U of DE Brown Lab
PROBHD    Z824801_0171 (
PULPROG               jmod
TD                   65536
SOLVENT              CDCl3
NS                    1024
DS                       4
SWH              23809.524 Hz
FIDRES            0.726609 Hz
AQ               1.3762560 sec
RG                     101
DW                  21.000 usec
DE                    6.50 usec
TE                   298.2 K
CNST2          145.0000000
CNST11           1.0000000
D1              5.00000000 sec
D20             0.00689655 sec
TD0                      1
SFO1           100.6228298 MHz
NUC1                   13C
P1                   10.00 usec
P2                   20.00 usec
PLW1           44.54999924 W
SFO2           400.1316005 MHz
NUC2                    1H
CPDPRG[2           waltz65
PCPD2                90.00 usec
PLW2           16.44099998 W
PLW12           0.20298000 W

F2 - Processing parameters
SI                   32768
SF             100.6127685 MHz
WDW                     EM
SSB                      0
LB                    1.00 Hz
GB                       0
PC                    1.40
```
